# Supplementary material for: Comparative and Functional Analyses Reveal Conserved and Variable Regulatory Systems That Control Lasalocid Biosynthesis in Different Streptomyces Species
Source: Microbiol Spectr. 2023 Feb 27;11(2):e03852-22. doi: 10.1128/spectrum.03852-22 (PMC10100954; doi:10.1128/spectrum.03852-22)
Supplement: Supplemental file 1 — Supplemental material. Download spectrum.03852-22-s0001.pdf, PDF file, 0.6 MB [file spectrum.03852-22-s0001.pdf]

Table S1 Strains used in this study

**Strains**	**Descriptions**	**Reference/source**
***E. coli* strains**		
E. coli Top 10	For plasmid propagation	Invitrogen
E. coli ET12567/pUZ8002	For conjugal transfer to *Streptomyces*	(1)
E. coli BL21(DE3)	For protein expression	Invitrogen
***Streptomyces* strains**		
S. sp. FXJ1.172	Wild-type lasalocid producer	This study
Δ*lodE*	*lodE* disruptive in-frame deletion mutant	This study
Δ*lodR1*	*lodR1* disruptive in-frame deletion mutant	This study
Δ*lodR2*	*lodR2* disruptive in-frame deletion mutant	This study
Δ*lodR3*	*lodR3* disruptive in-frame deletion mutant	This study
Δ*lodE*C	*lodE* complemented strain of Δ*lodE*	
Δ*lodR1*C	*lodR1* complemented strain of Δ*lodR1*	This study
Δ*lodR2*C	*lodR2* complemented strain of Δ*lodR2*	This study
Δ*lodR3*C	*lodR3* complemented strain of Δ*lodR3*	This study
S. lasalocidi ATCC 31180	Wild-type lasalocid producer	This study
Δ*las2*	*las2* disruption mutant	This study
Δ*las2*C	*las2* complemented strain of Δ*las2*	This study
*las2*OE	over-expression strain of *las2* in wild-type	This study
Δ*las3*	*las3* disruption mutant	This study
Δ*las3*C	*las3* complemented strain of Δ*las3*	This study
Δ*las3*-152-*las4*OE	*las4* was overexpressed in the *las3* disruption mutant via a vector pSET152::P$_{kasO*}$*las4*	This study
Δ*las3*-1139-*las4*OE	*las4* was overexpressed in the *las3* disruption mutant via a vector pKC1139::P$_{kasO*}$*las4*	This study
Δ*las4*	*las4* disruption mutant	This study
Δ*las4*C	*las4* complemented strain of Δ*las4*	This study
*las4*OE	over-expression strain of *las4* in wild-type	This study
31180-KABC	*S. lasalocidi* ATCC31180/pSET152::P$_{kasO*}$*lodA–C*; Apr^r	This study
31180-R1ABC	*S. lasalocidi* ATCC31180/ pSET152::*lodR1–C*; Apr^r	This study
S. coelicolor M1146	Derivative of *S. coelicolor* M145, Δ*act* Δ*red* Δ*cpk* Δ*cda;* used as a vector strain for *gusA* transcriptional fusion assays.	(2)
MP$_C$	*S. coelicolor* M1146/pIJ10500::P$_C$-*gusA*; Hyg^r	This study
MP$_{R3}$	*S. coelicolor* M1146/pIJ10500::P$_{R3}$-*gusA*; Hyg^r	This study

MP$_G$	*S. coelicolor* M1146/pIJ10500::P$_G$-*gusA*; Hyg^r	This study
MP$_H$	*S. coelicolor* M1146/pIJ10500::P$_H$-*gusA*; Hyg^r	This study
MP$_Q$	*S. coelicolor* M1146/pIJ10500::P$_Q$-*gusA*; Hyg^r	This study
MP$_S$	*S. coelicolor* M1146/pIJ10500::P$_S$-*gusA*; Hyg^r	This study
MP*c*(+)	*S. coelicolor* M1146/pIJ10500::P$_C$-*gusA*/pSET152::P$_{kasO*}$-*lodR3*; Apr^r, Hyg^r	This study
MP$_{R3}$(+)	*S. coelicolor* M1146/pIJ10500::P$_{R3}$-*gusA*/pSET152::P$_{kasO*}$-*lodR3*; Apr^r, Hyg^r	This study
MP$_G$(+)	*S. coelicolor* M1146/pIJ10500::P$_G$-*gusA*/pSET152::P$_{kasO*}$-*lodR3*; Apr^r, Hyg^r	This study
MP$_H$(+)	*S. coelicolor* M1146/pIJ10500::P$_H$-*gusA*/pSET152::P$_{kasO*}$-*lodR3*; Apr^r, Hyg^r	This study
MP$_Q$(+)	*S. coelicolor* M1146/pIJ10500::P$_Q$-*gusA*/pSET152::P$_{kasO*}$-*lodR3*; Apr^r, Hyg^r	This study
MP$_S$(+)	*S. coelicolor* M1146/pIJ10500::P$_S$-*gusA*/pSET152::P$_{kasO*}$-*lodR3*; Apr^r, Hyg^r	This study

Table S2 Plasmids used in this study

Plasmids	Descriptions	Reference/source
pKC1139	Multiple-copy, temperature-sensitive, *E. coli*-*Streptomyces* shuttle vector, Apr^r	(3)
pSET152	Integrative *E. coli*-*Streptomyces* shuttle vector Apr^r	(4)
pIJ10500	A derivative of pMS82 containing φBT1 integrase coding gene, Hyg^r	(5)
pUC119::*neo*	Source of *neo* gene conferring kan^r	(6)
pET23b	Vector for His$_6$-tagged protein expression in *E. coli* Amp^r	Novagen
pET28a	Vector for His$_6$-tagged protein expression in *E. coli*, kan^r	Novagen
pKC1139::*lodE*	*lodE* disruptive in-frame deletion	This study
pKC1139::*lodR1*	*lodR1* disruptive in-frame deletion	This study
pKC1139::*lodR2*	*lodR2* disruptive in-frame deletion	This study
pKC1139::*lodR3*	*lodR3* disruptive in-frame deletion	This study
pKC1139::*las2*::*neo*	*las2* disruption	This study
pKC1139::*las3*::*neo*	*las3* disruption	This study
pKC1139::*las4*::*neo*	*las4* disruption	This study
pKC1139::P$_{kasO*}$*las4*	*las4* over expression	This study
pSET152::P$_{kasO*}$*lodE*C	*lodE* complementation	This study
pSET152::P$_{kasO*}$*lodR1*C	*lodR1* complementation	This study
pSET152::P$_{kasO*}$*lodR2*C	*lodR2* complementation	This study
pSET152::P$_{kasO*}$*lodR3*C	*lodR3* complementation and over expression	This study
pSET152::P$_{kasO*}$*las2*C	*las2* complementation and over expression	This study
pSET152::P$_{kasO*}$*las3*C	*las3* complementation	This study
pSET152::P$_{kasO*}$*las4*C	*las4* complementation and over expression	This study
pSET152::*lodR1ABC*	Heterologous expression of *lodR1–C*	This study
pSET152::P$_{kasO*}$*lodABC*	Heterologous expression of *lodA–C*	This study
pIJ10500::P$_C$-*gusA*	pIJ10500 derivative with insertion of P$_C$ and *gusA*	This study
pIJ10500::P$_{R3}$-*gusA*	pIJ10500 derivative with insertion of the intergenic region of *lodR2–lodR3* and *gusA*	This study
pIJ10500::P$_G$-*gusA*	pIJ10500 derivative with insertion of P$_G$ and *gusA*	This study
pIJ10500::P$_H$-*gusA*	pIJ10500 derivative with insertion of P$_H$ and *gusA*	This study
pIJ10500::P$_Q$-*gusA*	pIJ10500 derivative with insertion of P$_Q$ and	This study

	gusA	
pIJ10500::P_S-*gusA*	pIJ10500 derivative with insertion of P_S and *gusA*	This study
pET23b::*lodR1*	LodR1 expression vector	This study
pET23b::*lodR2*	LodR2 expression vector	This study
pET23b::*lodR3*	LodR3 expression vector	This study
pET28a::*las3*	Las3 expression vector	This study

Table S3 Primers used in this study

**Primers**	**Sequence (5’-3’)[a]**	**Purpose**
lodR1-L-F	***GATCCGCGGCCGCGCGCGAT***GCAACGCTC ATCAGGAACGCC	*lodR1* disruptive in-frame deletion
lodR1-L-R	CGGCTCGCCATGCTCTGACAC	
lodR1-R-F	***GTGTCAGAGCATGGCGAGCCG***CTGTTTGC GTTCCTGTACCGACAT	
lodR1-R-R	***GACATGATTACGAATTCGAT***GCCGTCATCG CCTACGAAGTC	
lodR1-tF	TGGACGCCTACCAACAGGAGCTG	Verification of *lodR1* disruptive in-frame deletion
lodR1-tR	GGAGGGCATTGAGGATCGGATTGA	
lodR1-orf-F	***CGTGCAGGACTGGGGGAGTT***ATGTCGGTA CAGGAACGCAAAC	*lodR1* complementation
lodR1-orf-R	***GACATGATTACGAATTCGAT***TCAGAGCATG GCGAGCCG	
lodR2-L-F	***GATCCGCGGCCGCGCGCGAT***GGGCGGCGT GAAACCCCTATT	*lodR2* disruptive in-frame deletion
lodR2-L-R	CGCGAAGCGCTCGATGAACCT	
lodR2-R-F	***AGGTTCATCGAGCGCTTCGCG***TGGCGACA ACAGGAGGCAAG	
lodR2-R-R	***GACATGATTACGAATTCGAT***CACCTGGAGG AACCGCTCTATCA	
lodR2-tF	GACAGCGACAGCGGGAAGATGA	Verification of *lodR2* disruptive in-frame deletion
lodR2-tR	GTGCCCTATTGCGGGAGAACG	
lodR2-orf-F	***CGTGCAGGACTGGGGGAGTT***GTGACCGAC AAGCCCGC	*lodR2* complementation
lodR2-orf-R	***GACATGATTACGAATTCGAT***GGCGTCAGTT GCTTGCCTC	
lodR3-L-F	***GATCCGCGGCCGCGCGCGAT***CATCGGGAC GTTGATGAGGA	*lodR3* disruptive in-frame deletion
lodR3-L-R	ACCTAACGGTGTGCTGCTGC	
lodR3-R-F	***GCAGCAGCACACCGTTAGGT***CACGAGAAC GACTGCCAGTCCAT	
lodR3-R-R	***GACATGATTACGAATTCGAT***GGATTCATCG TCAACGCCCTG	
lodR3-tF	CGCATTCGAGGAAGTGGTGTCC	Verification of *lodR3* disruptive in-frame deletion
lodR3-tR	GCTCCAGGAAGGTGAAGGTGGG	
lodR3-orf-F	***CGTGCAGGACTGGGGGAGTT***ATGGAATTC TTAGAACAACGAACAG	*lodR3* complementation and overexpression
lodR3-po-R	***GACATGATTACGAATTCGAT***CTATGTGGCG	

	ATGGACTGGCA	
lodE-L-F	***GATCCGCGGCCGCGCGCGAT***TGTGACCGCTCAACGGGGACC	*lodE* disruptive in-frame deletion
lodE-L-R	GTCGCCAAGAAGCTGCACTCGAC	
lodE-R-F	***GTCGAGTGCAGCTTCTTGGCGAC***GAAGACGAGGTTGTACGCGTTGG	
lodE-R-R	***GACATGATTACGAATTCGAT***GCGACAGCGACTGGGTGAAGAG	
lodE-tF	GACGAAGGAGTCCTGCACATTGGA	Verification of *lodE* disruptive in-frame deletion
lodE-tR	TCGCCTCCCTCTGGTTGTTGC	
lodE-orf-F	***CGTGCAGGACTGGGGGAGTT***ATGGATGCCGACATGGCC	*lodE* complementation
lodE-orf-R	***GACATGATTACGAATTCGAT***CGGTGACGCTAACCAAGGAG	
neo-Gib-F	***AGATCT***ATCCCCTGGATACCGCTCGCCGCAG	*neo* amplification for Gibson assembly
neo-Gib-R	***AGATCT***TACCCGAACCCCAGAGTCCCG	
PkasO*-F	***GATCCGCGGCCGCGCGCGAT***TGTTCACATTCGAACGGTCTCTGCT	P_{kasO*} amplification for Gibson assembly
PkasO*-R	AACTCCCCCAGTCCTGCACGC	
lodR1ABC-F	***GATCCGCGGCCGCGCGCGAT***CGCAGGCTTCAACTTCCCCACCT	Heterologous expression of *lodR1–C*
lodR1ABC-R	***GACATGATTACGAATTCGAT***GCCATTTCTACCTCACCACCCACCA	
lodABC-F	***CGTGCAGGACTGGGGGAGTT***ATGCTCAGCGCGCTCGAGGTCGTCA	Heterologous expression of *lodA–C*
lodABC-R	***GACATGATTACGAATTCGAT***GGTGACCGTGCTGGTGGGCGACA	
las2-L-F	***GATCCGCGGCCGCGCGCGAT***ACTGTTATCGCCAGGGAATATGGCGC	*las2* disruption
las2-L-R	***GGTATCCAGGGGATAGATCT***CATGAACGTGATGCTGGTCGTCTGC	
las2-R-F	***CTGGGGTTCGGGTAAGATCT***GGCGAAGACCAGGTTGTAGGCG	
las2-R-R	***GACATGATTACGAATTCGAT***CGACTGGGTGAACAGGGTGAGAATC	
las2-tF	CCGTGCGGTACTGCCGTATCCA	Verification of *las2* disruption
las2-tR	GGCACATCAGAAACAACCGAAGGAT	
las2-OE-F	***CGTGCAGGACTGGGGGAGTT***ATGAGCGTCTCGGATGCCCGAC	*las2* complementation and over expression
las2-OE-R	***GACATGATTACGAATTCGAT***GCGGTCGAG	

	GGGGACGCTAGAG	
las3-L-F	***GATCCGCGGCCGCGCGCGAT***CAAGGAGCGGGTGGCGTGTAAC	*las3* disruption
las3-L-R	***GGTATCCAGGGGATAGATCT***ACCCTGCGTCGCCTCCTTCACT	
las3-R-F	***CTGGGGTTCGGGTAAGATCT***GCAACGTCTGGTACGAGCTCATGCG	
las3-R-R	***GACATGATTACGAATTCGAT***ACTCCGCCAGGTACAGGCTCTCCG	
las3-tF	TCCTTCGGTTGTTTCTGATGTGCC	Verification of *las3* disruption
las3-tR	GGAGCGTGATGGACGTGGAGA	
las3-OE-F	***CGTGCAGGACTGGGGGAGTT***GTGACCGAGAAGCCGACGCAG	*las3* complementation
las3-OE-R	***GACATGATTACGAATTCGAT***GACTGGCAAATGCCTGACGACG	
las4-L-F	***GATCCGCGGCCGCGCGCGAT***CGGTGAACAGCAGTCCGAACAGG	*las4* disruption
las4-L-R	***GGTATCCAGGGGATAGATCT***AGGTGGAGGAGGCAGTGCAAGGA	
las4-R-F	***CTGGGGTTCGGGTAAGATCT***GCCCACGACTGCTCACCAAGG	
las4-R-R	***GACATGATTACGAATTCGAT***GTGCTGGGCATGCACTTCTTCAAC	
las4-tF	GGAACACCGCTCGGCGTCGTA	Verification of *las4* disruption
las4-tR	GAGCAGTCGTGGGCGCACCTT	
las4-OE-F	***CGTGCAGGACTGGGGGAGTT***GTGCCCTTCTCTGTCATGGAATCCG	Complementation and overexpression of *las4*
las4-OE-R	***GACATGATTACGAATTCGAT***CCTACGTGGCGATGGACTGGCA	
neo-Gib-F	AGATCTATCCCCTGGATACCGCTCGCCGCAG	*neo* amplification for Gibson assembly
neo-Gib-R	AGATCTTACCCGAACCCCAGAGTCCCG	
lodA-BF	TCGCTCGTCCTCGCCGTCATCT	Co-transcriptional analysis of the genes in *lod* by RT-PCR
lodA-BR	ACTGTCCGCCGTTCCCTTGTGC	
lodB-lodCF	AGTTCATGAAGGCTCAACCGGGATT	
lodB-lodCR	AGGTCCGATCCGACGATTCCGT	
lodD-lodEF	GCGAGGCGGAGGGCGACTTCGTA	
lodD-lodER	CATCGGCGGCATCATCGTGGG	
lodR2-lodR3F	CGCTGCCCTTCTTCGAGTTCATGCA	
lodR2-lodR3R	GACGGCGAGATTCCGCACGAT	
lodR3-lodFF	GCGACATATCGACCAAGCTCTACATCA	
lodR3-lodFR	TCCTTCCAGAAGCGGTAGCCCTC	
lodH-lodIF	CGTCCTCCTCCCCGCCATCAA	

lodH-lodIR	ATCAACCCGCCCCAGCGAAGC	
lodI-lodJF	CAGCTCCGCAACCGCCTCAAC	
lodI-lodJR	TCTCCCAGGCGACCTCCAGCA	
lodJ-lodKF	ACGGTGGCGGTGGAGGAGATGG	
lodJ-lodKR	CTTGACGTAGGTCTTGCCGGGAGC	
lodK-lodLF	CCGTGTTCGGTGAACTCGACAACCT	
lodK-lodLR	GCCAACGCGACAGAGCACTCCTC	
lodL-lodMF	CCGCCGCACTCCACAACGCA	
lodL-lodMR	TCGGTCCAATCGATCCGGTACAGGT	
lodM-lodNF	CGGTCATCTGCTGGAGACGCTGG	
lodM-lodNR	TCGGTCCAATCGATCCGGTACAGGT	
lodN-lodOF	CCGCGCCTTCAAGGAGCTGGGGTTC	
lodN-lodOR	CGTCGGTCACCTGTGCGCCGTAGCC	
lodO-lodPF	GGCGCTCATGTCCCTGTCCCTT	
lodO-lodPR	CGCTGCCTGCTGAACTCGGTGAA	
lodS-lodTF	CCGGTCTACGGCCACGGTATGAG	
lodS-lodTR	GAAGCGGATGCGGGGCGAGTA	
27F	AGAGTTTGATCCTGGCTCAG	
1492R	TACGACTTAACCCCAATCGC	
las1-las2F	TGGTGGGTCGTCAGGTAGAAGTGGC	Co-transcriptional analysis of the genes in *las* by RT-PCR
las1-las2R	CATGAACGTGATGCTGGTCGTCTGC	
las3-las4F	GTCGAGACCCTCGGCAGGGAC	
las3-las4R	AGGTTTTGGCCGGCGGTTGC	
las4-las5F	AAGGTGCGCCCACGACTGCT	
las4-las5R	ACCGGACCAGCTCGGCCTTGC	
lasAI-lasAIIF	AAATGGAAGGAAGCGCAGAACTCTGCCG	
lasAI-lasAIIR	CCACAACGGCTGCTCCGTGGGC	
lasAII-lasAIIIF	CTCATCGACCGGGACCTCGGACTG	
lasAII-lasAIIIR	GTTGCGGAGCGCCTGGCGGA	
lasAIII-lasAIVF	GCGGGCCTTCAAGGACCTGGGCT	
lasAIII-lasAIVR	CACACCGGTCGCGGTTCCCCTTTG	
lasAIV-lasAVF	GACACCGACACCCGCGTGAAGA	
lasAIV-lasAVR	TCCTGCCGCAGCGCCCGGCAG	
lasAV-lasAVIF	CACGGGCGAGACGGCTGAGAAC	
lasAV-lasAVIR	CTGCGGGTCCATCGCGAGGGCC	
lasAVI-lasAVIIF	ACGCAGGCGGCGAACGTCCT	
lasAVI-lasAVIIR	CTCGCGGGGCGAGATGCCGAAC	
lasC-lasBF	AGGCGGCTGCGACGAACTT	
lasC-lasBR	AGGTAGTCCATCGTCGCGGAGACC	
lodR1 RT F	TGCAGGGACGCCCAGAACACC	RT-qPCR analyses of *lod* genes
lodR1 RT R	CCCGGAACCTCTCAAGGACGCTTT	
lodA RT F	TCAATGCCCTCCCGGAGGAAAGC	
lodA RT R	AGGACGAGCGAGCCCATGTAC	

lodC RT F	TGTACTCGCGCATCATCGATCCGTC	
lodC RT R	AAGCCGGTCAGGTCCGATCCG	
lodE RT F	TAGGCGTTGGTGAGCACCGTG	
lodE RT R	ACTGCCGACCGAGCGCAGT	
lodR2 RT F	TGGCCGAGCAGCTTCAGGTGAG	
lodR2 RT R	TGTCGACCCGGAAGTGATCCTGC	
lodR3 RT F	TTCCGGTGGTGGTGAGTGTCGATG	
lodR3 RT R	GTAATTCGGTGTCGAACGTGGGGT	
lodF RT F	TACCACATACTGGGTTCGGACCTGT	
lodF RT R	TCGACCGACAGACAGTGGGCCA	
lodG RT F	TTGGAAGGGGCCCATCGGGAAT	
lodG RT R	TCAACCGGGCGGTGGGCAT	
lodH RT F	TGATCGGCCTGTCCTGCCGTTTT	
lodH RT R	GCCAGCGTTCCTCGGGGACA	
lodL RT F	TACGCCTTCGGTCTGGAGGGACC	
lodL RT R	CGACAGAGCACTCCTCCTGCCG	
lodP RT F	TGACCGGCTCCGCAGCGGAT	
lodP RT R	ATGCCTGCTCTCGAGCATGGTGAA	
lodQ RT F	TCGAGGTGGGAGTTGCTTTCCGC	
lodQ RT R	ACCAACTCCCGGCTCATCGAATT	
lodS RT F	TGCGCAAGGGAGTTCCGCAG	
lodS RT R	ACACCTGGCCGTCGGGAATTCC	
q16S F	GCAATCTGCCCTGCACTCTG	
q16S R	AGCCGTTACCTCACCAACAAGC	
las1 RT F	TGCGCAAGGGAGTTCCGCAG	RT-qPCR analyses of *las* genes
las1 RT R	ACACCTGGCCGTCGGGAATTCC	
las2 RT F	TGGACGAACGACTCCTGCACGCTGG	
las2 RT R	CGCTCCTCGGCACCATCCTGACCTC	
las3 RT F	TGCGCAAGGGAGTTCCGCAG	
las3 RT R	ACACCTGGCCGTCGGGAATTCC	
las4 RT F	ATCGAGCGGTTCCTGCGGGTGT	
las4 RT R	CGGTCGTGGTGGATGAGCGACTTG	
las6 RT F	CCGTCATCACCGTGTCGATCTCGT	
las6 RT R	CGCCCTGCTCTTCCCTTACCTCAA	
lasAI RT F	TACAGGCGACCGAGGACGAGGTGC	
lasAI RT R	TGGAAGGCGTGGCTGACGTGGAG	
lasAV RT F	TCGACCACGTCCTGCGACCCAA	
lasAV RT R	ATGCCTGCGGCGGAGGAGAAGA	
lasAVII RT F	GCCACGAGGTACTGCCACAGACACTG	
lasAVII RT R	GTGCCGCTGATGCCGAAGGAG	
las7 RT F	TTCCGTCGAGGTGGGAGTTGC	
las7 RT R	CAGTATTTCTCCTACCGCGTGCTGA	
lasC RT F	CCAGTTCATGATCGCCTGCAGTCGT	

lasC RT R	CAGCGCCAGCACCTCCGTCTTCT	
31180-16S-F	CCACCTTCCTCCGAGTTGACC	
31180-16S-R	GCTCGTGTCGTGAGATGTTGGGT	
R1-A F	GAGTTGGCCGCTTTCCTCCGG	*lodR1–lodA* probe for EMSA and DNase I footprinting
R1-A R	GGACGGGCTGGCTGTATTCGA	
R2-E F	GGCTGGTCGAGGCCTGCAGAT	*lodR2–lodE* probe for EMSA and DNase I footprinting
R2-E R	CGGGGCTCACCTGAAGCTGCT	
las2-3F	GCATCCGCGGGAAGCCCG	*lod2–lod3* probe for EMSA and DNase I footprinting
las2-3R	GCAGGGCGACGTTCAGGACC	
R1pro F	***AATCATAT***GTCGGTACAGGAACGCAAACA	Expression of LodR1 protein with pET23b
R1pro R	***AATCTCGAG***GAGCATGGCGAGCCGGTCCA	
R2pro F	***AATCATATG***ACCGACAAGCCCGCGCA	Expression of LodR2 protein with pET23b
R2pro R	***AATCTCGAG***GTTGCTTGCCTCCTGTTGTCGCC	
Las2pro F	***TGCCGCGCGGCAGCCATATG***GTGACCGAGAAGCCGACGCAGGC	Expression of Las2 protein with pET23b
Las2pro R	***GTGGTGGTGGTGGTGCTCGA***GGAATTTCCCTCCTGCTGACGCCAG	
gusA-F	ATGACCGGTCTGCGGCCC	Construction of *gusA* transcriptional fusion systems
gusA-R	***GATCATATGAGGCCTCTCGA***TCACTGCTTCCCGCCCTGC	
LodB-CF	***CGTCGTGGTCCTTGTAGTCC***GCACAAGGGAACGGCGGACAGT	
LodB-CR	***ACGGGCCGCAGACCGGTCAT***AGGCTGCTCCCAGTCGATGGCC	
LodR2-R3F	***CGTCGTGGTCCTTGTAGTCC***GCGACAACAGGAGGCAAGCAAC	
LodR2-R3R	***ACGGGCCGCAGACCGGTCAT***CACTTCCTCGAATGCGGCTTGCA	
LodG-HF	***CGTCGTGGTCCTTGTAGTCC***ATGGCCGCGTCGATGCCCTCCT	
LodG-HR	***ACGGGCCGCAGACCGGTCAT***ATGCCCTCCCGCATTCCCCGTACTC	
LodH-GF	***CGTCGTGGTCCTTGTAGTCC***GCCTCCCAGTCCCAGCACATACG	
LodH-GR	***ACGGGCCGCAGACCGGTCAT***CGAGTCCTTTCGTCAATGGACCGTTG	
LodQ-SF	***CGTCGTGGTCCTTGTAGTCC***AAGACCATCTTTCCGCCGGGACGCA	

LodQ-SR	***ACGGGCCGCAGACCGGTCAT***CGGTTCACG CGATCCTTTCCCGC	
LodS-QF	***CGTCGTGGTCCTTGTAGTCC***AGTTGGTGT CGTCGGGTGCCGCC	
LodS-QR	***ACGGGCCGCAGACCGGTCAT***CGGTTCACG CGATCCTTTCCCG	
gusAT-F	CAGGCTTTACACTTTATGCTTCCG	*gusA* amplification for Gibson assembly
gusAT-R	TCTTCCTTGACTCCGTCATGGTC	

[a] Gibson assembly homologous sequences matching the right or left end of the kanamycin resistance gene cassette, P_{kasO*} promoter, pKC1139, pSET152 and other vectors are in bold italics.

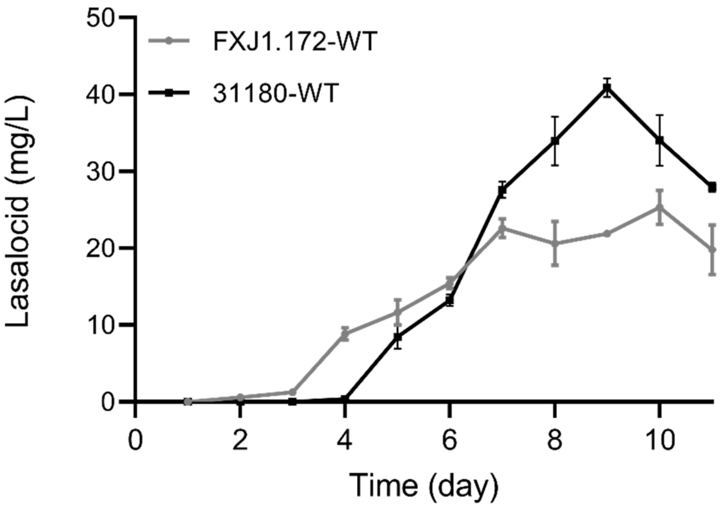

Fig S1 Time course of lasalocid production in *Streptomyces* sp. FXJ1.172 and *Streptomyces lasalocidi* ATCC 31180^{T}. Error bars show the standard deviation of three independent experiments.

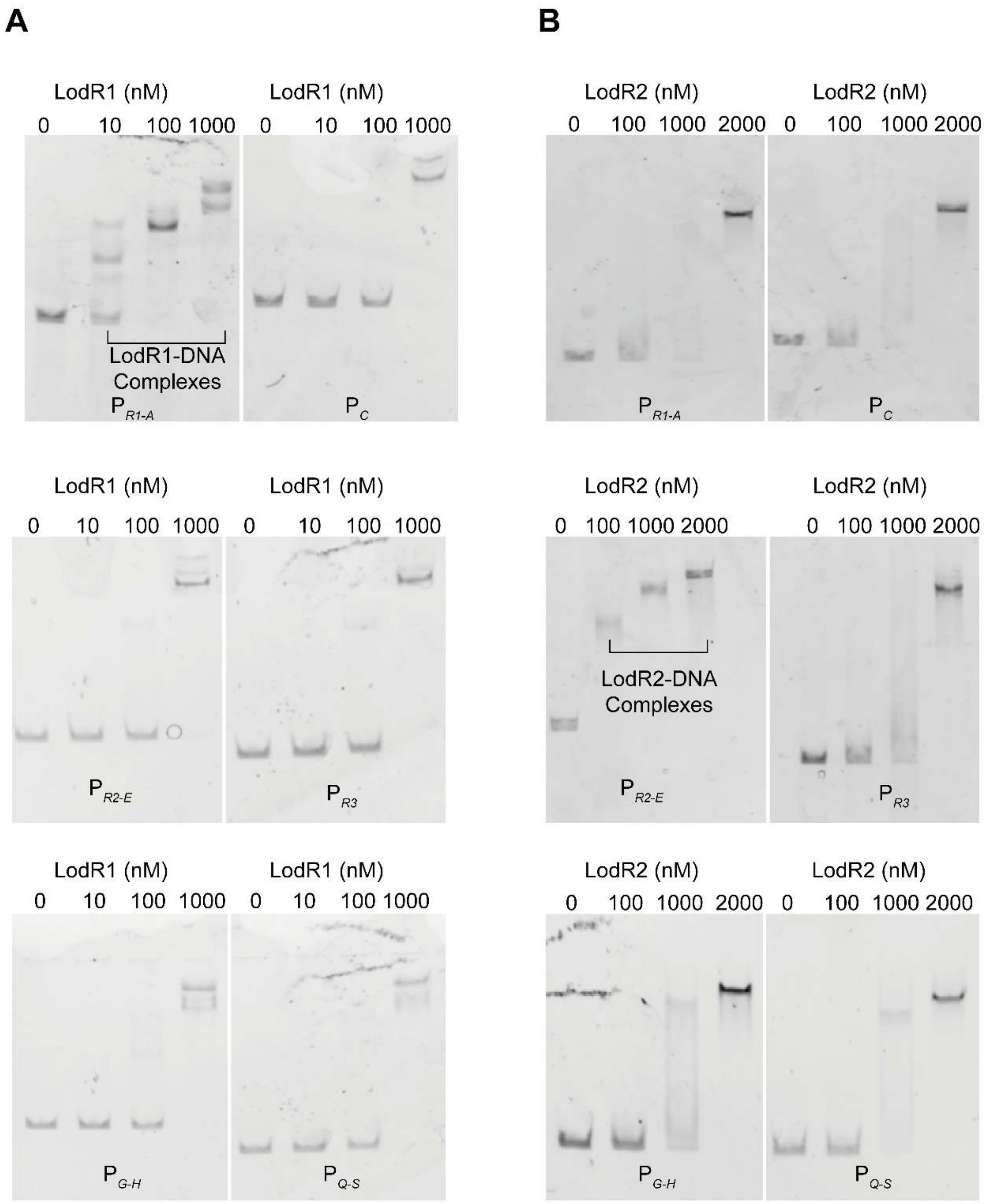

Fig S2 EMSA assays of LodR1 and LodR2 with all potential promoters indicated in Fig. 3A and with the intergenic region of *lodR2–lodR3*. A, LodR1 specifically binds to the intergenic region of *lodR1–lodA*. B, LodR2 specifically binds to the intergenic region of *lodE–lodR2*. Non-specific bindings were observed when the proteins were overloaded.

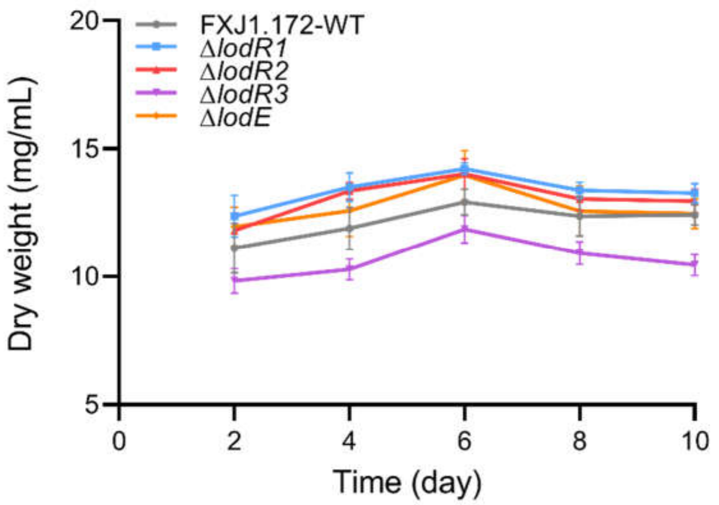

Fig S3 Growth curves of *Streptomyces* sp. FXJ1.172 wild type strain and its derivatives grown in SSC liquid culture. WT, wild type; Δ*lodR1*, *lodR1* disruption mutant; Δ*lodR2*, *lodR2* disruption mutant; Δ*lodR3*, *lodR3* disruption mutant; and Δ*lodE*, *lodE* disruption mutant. Error bars show the standard deviation of three independent experiments.

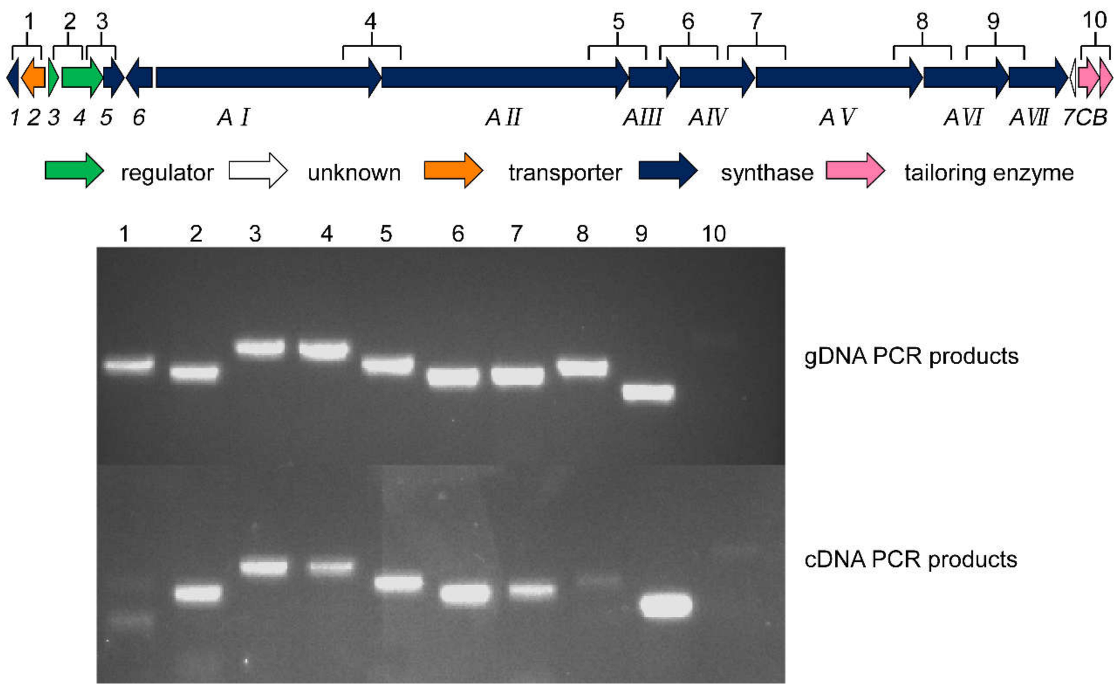

Fig S4 Co-transcriptional analysis of the genes in the *las* cluster by RT-PCR. The regions used for PCR amplification are labelled 1–10. Total RNA of *S. lasalocidi* ATCC 31180^{T} WT was isolated after incubation for 120 h and used for synthesizing cDNA as the template. The genomic DNA (gDNA) was used as a positive control of the template.

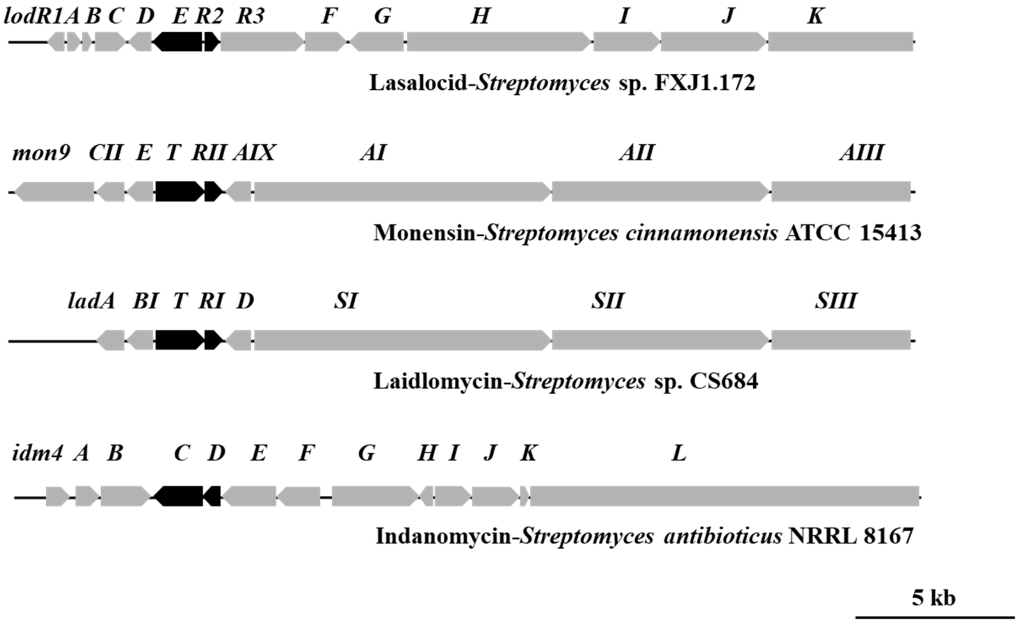

Fig S5 Organizations of the *lodE* and *lodR2* homologs in other polyether ionophore biosynthetic gene clusters (partial). The *lodE*–*lodR2* homologs and other genes are indicated by black and light grey arrows, respectively.

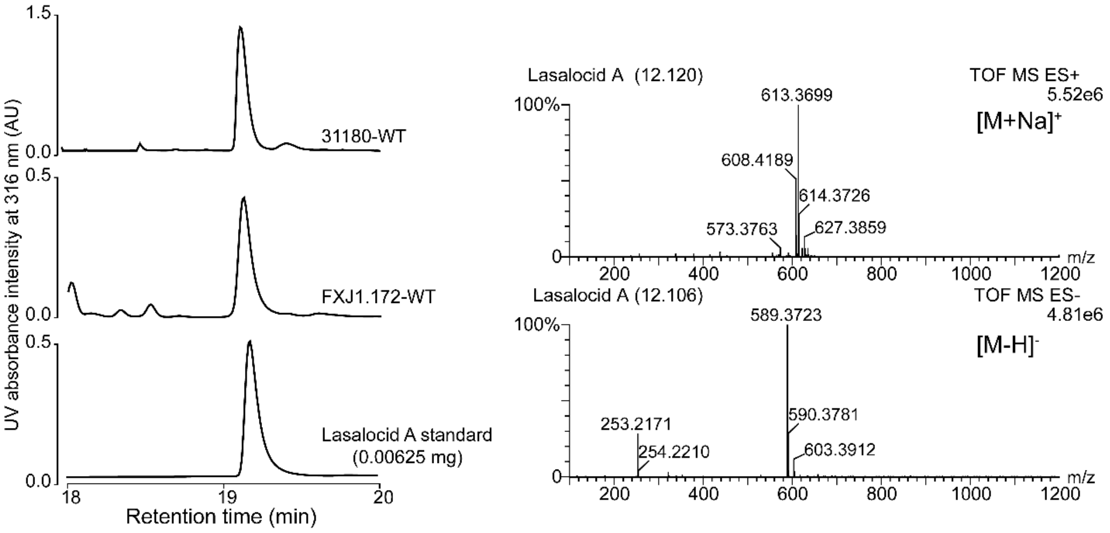

Fig S6 HPLC profiling and mass spectrum of lasalocid A. The exact mass of lasalocid A is 590.3818.

References

1. Macneil DJ, Gewain KM, Ruby CL, Dezeny G, Gibbons PH, Macneil T. 1992. Analysis of *Streptomyces avermitilis* genes required for avermectin biosynthesis utilizing a novel integration vector. Gene 111:61-68.
2. Gomez-Escribano JP, Bibb MJ. 2011. Engineering *Streptomyces coelicolor* for heterologous expression of secondary metabolite gene clusters. Microb Biotechnol 4:207-215.
3. Kieser T, Bibb MJ, Buttner MJ, Chater KF Hopwood DA. 2000. Practical Streptomyces Genetics. John Innes Centre, Norwich Research Park, Colney, Norwich NR4 7UH, England: John Innes Foundation.
4. Bierman M, Logan R, O'Brien K, Seno ET, Rao RN, Schoner BE. 1992. Plasmid cloning vectors for the conjugal transfer of DNA from *Escherichia coli* to *Streptomyces* spp. Gene 116:43-49.
5. Gregory MA, Till R, Smith MCM. 2003. Integration site for *Streptomyces* phage phi BT1 and development of site-specific integrating vectors. J Bacteriol 185:5320-5323.
6. Pan Y, Liu G, Yang H, Tian Y, Tan H. 2009. The pleiotropic regulator AdpA-L directly controls the pathway-specific activator of nikkomycin biosynthesis in *Streptomyces ansochromogenes*. Mol Microbiol 72:710-723.
